# Antarctic Fungi as a Source of Alternative Antifungal Compounds: Bioactive Metabolites from South Shetland Islands Fungi with Activity Against *Candida* Species

**DOI:** 10.3390/microorganisms14030617

**Published:** 2026-03-10

**Authors:** Nicole Cortez, Muhammad Javid Iqbal, Cecilia Villegas, Jaime R. Cabrera-Pardo, Viviana Burgos, Sigisfredo Garnica, Sarah Zuern, Marcelo Ortega-Silva, Cristian Paz

**Affiliations:** 1 Laboratory of Natural Products & Drug Discovery, Center CEBIM, Department of Basic Sciences, Faculty of Medicine, Universidad de La Frontera, Temuco 4780000, Chile; n.cortezsalvo01@gmail.com (N.C.); m.iqbal01@ufromail.cl (M.J.I.); 2 Departamento de Ciencias Biológicas y Químicas, Facultad de Recursos Naturales, Universidad Católica de Temuco, Rudecindo Ortega, Temuco 4780000, Chile; cecilia.villegas@uct.cl; 3 Laboratorio de Química Aplicada y Sustentable (LabQAS), Departamento de Química, Universidad del Bío-Bío, Avenida Collao 1202, Concepción 4051381, Chile; jacabrera777@gmail.com; 4 Escuela de Tecnología Médica, Facultad de Salud, Universidad Santo Tomás, Temuco 4780000, Chile; vburgos7@santotomas.cl; 5 Laboratorio de Micología, Instituto de Bioquímica y Microbiología, Universidad Austral de Chile, Valdivia 5090000, Chile; sigisfredo.garnica@uach.cl (S.G.); sarah.zuern@uach.cl (S.Z.); 6 Escuela de Tecnología Médica, Facultad de Ciencias, Universidad San Sebastián, Lago Panguipulli 1390, Puerto Montt 5501842, Chile

**Keywords:** antarctic fungi, *Candida* species, alternative antifungal compounds, psychrophilic microorganisms

## Abstract

The emergence of drug-resistant *Candida* species has intensified efforts to discover novel bioactive compounds. Antarctic environments harbor psychrophilic microorganisms that produce unique secondary metabolites adapted to extreme conditions, making them valuable natural resources for drug discovery. During the 2020 Antarctic Scientific Expedition, we collected 19 sediment samples from the South Shetland Islands and isolated 14 fungal strains belonging to *Cladosporium*, *Oidiodendron*, *Penicillium*, *Pseudeurotium*, and *Pseudogymnoascus* genera. Total organic extracts obtained from 21-day cultures were evaluated for antimicrobial activity against pathogenic yeasts and bacteria. *Oidiodendron* sp. (ECA57-20) and *Pseudogymnoascus* sp. (ECA57-61) demonstrated strong anti-*Candida* activity with minimum inhibitory concentrations ranging from 7.81 to 62.5 µg/mL against *C. albicans*, *Pichia kudriavzevii* (*C. krusei*), *C. tropicalis*, *Nakaseomyces glabratus* (*C. glabrata*), and *Clavispora lusitaniae* (*C. lusitaniae*). GC-MS (gas chromatography mass spectrometry) metabolomic profiling suggests a broad diversity of secondary metabolites across active strains, which may contribute to the observed biological activities. These findings support the potential of Antarctic fungi as sources of alternative antifungal agents.

## 1. Introduction

The search for novel bioactive metabolites with pharmacological applications has become a pressing need, due to the appearing of new viruses (like SARS-CoV-2) and resistant microorganisms, like bacteria and yeast [1,2]. Each year in the United States, 35,000 deaths occur due to antibiotic-resistant infections [3]. Moreover, fungal infections affect over one billion people worldwide and result in more than 1.5 million deaths annually, with candidemia being its most common manifestation [4,5,6]. *Candida albicans* is the main cause of invasive candidiasis, but in recent decades, non-Candida species such as *Candida parapsilosis*, *Candida tropicalis*, *Nakaseomyces glabratus*, and *Pichia kudriavzevii* have become a global concern [7,8].

Natural products have been a key source of new drugs, accounting for more than 20% of pharmaceutical substances in the International Pharmacopeia [9]. Between 1981 and 2014, 70% of the 1562 drugs approved were of natural origin [10]. In 2019, 9 of the 38 FDA-approved drugs came from natural products [11]. In this context, fungi isolated from pristine Antarctic soils represent a novel source in the search for new bioactive molecules.

According to Arenz et al. [12] fungi have been part of Antarctic ecosystems for more than 200 million years. Fossil evidence suggests their presence in the region since at least the Triassic Period [13]. These organisms have been reported from different soil types and substrates, spanning geographically distant locations and a variety of habitats in Antarctica. The earliest documented records date back to the early 20th century [12].

Fungi in pristine soils are more diverse than previously thought and their richness, abundance, and composition are generally determined primarily by environmental conditions, among which soil parameters appear to be the most prominent influencing factors [14]. The microbial biodiversity of Antarctica is mainly concentrated in ice-free areas, which represent less than 1% of the continent. Collins Glacier, also known as Bellinghausen Dome, has been retreating due to regional warming and could disappear in about 285 years, providing unique habitats to study microbial succession, as well as to trigger the discovery of new species that are able to produce novel secondary metabolites and bioactive enzymes with biotechnological potential [15,16].

The list of non-lichenized fungi reported from Antarctic (including sub-Antarctic) regions is extensive, with more than 1000 species [12]. Most filamentous fungi are cosmopolitan species; some fungi are psychrophilic, even more are psychrotolerant [17]. Antarctic mycobiota exhibit a zonal distribution; for example, in Subantarctic regions, *Antarctomyces psychrotrophicus*, *Geomyces pannorum*, and *Exophiala* sp. are dominant groups of mycomycetes, while on the Antarctic Peninsula, *Geomyces pannorum*, *Thelebolus microspores*, and *Mortierella* spp. dominate, and *Cadophora luteo-olivacea*, *Cadophora malorum*, *Dioszegia* sp, *Geomyces pannorum*, *Mortierella alpina*, *Phoma herbarum*, and *Thelebolus microsporus* are prevalent in Victoria Land and McMurdo Dry Valleys, which have the most severe climatic conditions in the Antarctic region [18]. However, it is estimated that between 10 and 30% of total microbiota have been cultured [18,19].

Fungal cultures from pristine Antarctic soil samples have shown to be a new source of bioactive metabolites; for example, *Penicillium* sp. isolated near the Great Wall Station produce the alkaloids meleagrin, neoxalin, and chestiomycin A, exhibiting significant cytotoxic effects against K562, MCF-7, A549, U937, Hela, DU145, HL60, and HT29 cell lines, with IC_50_ values ranging from 2.73 to 17.7 μM [20]. Moreover, different Antarctic fungi such as *Aspergillus flavus*, *A. terreus*, *Oidiodendron truncatum*, *Penicillium chrysogenum*, and other *Penicillium* species have shown antifungal activity against *Candida albicans* [14].

In this study, we identify fungal strains from sediments collected in different locations of the South Shetland Islands, such as Collins Glacier, Artigas Base, Hanna Point, and Deception Island during the ECA 57 (Expedición Científica Antártica 57) by INACH (Instituto Antártico Chileno) in the summer of 2020, and we evaluate their biotechnological potential against pathogenic microorganisms. The aim of this study was to identify Antarctic fungi with biotechnological potential that generates bioactive metabolites against pathogenic bacteria and yeasts.

## 2. Materials and Methods

### 2.1. Sampling Collection

**Figure 1:**
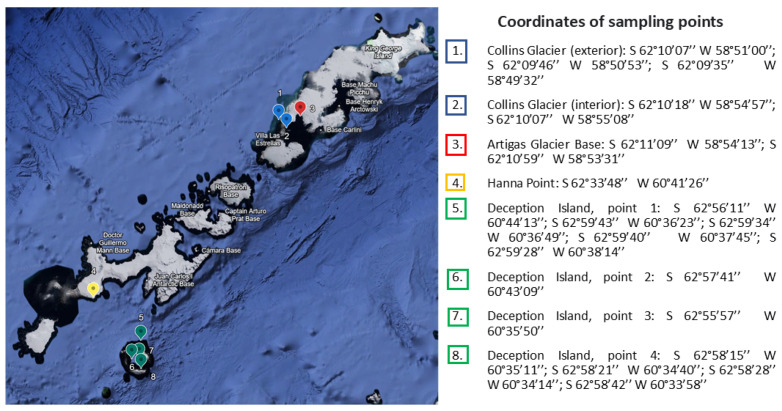
Sampling sites at the South Shetland Islands during the Antarctic expedition ECA-57; the figure was created using Google Earth web (https://earth.google.com/web/, accessed on 17 December 2025).

Sediments from South Shetland Islands were collected in January 2020 during the Antarctic campaign ECA-57 organized by the Instituto Antártico Chileno (INACH). Sampling points are summarized in Figure 1. In total, 19 soil samples (~10 g each) were collected: 5 from Collins Glacier, 2 from Artigas station, 11 from Deception Island, and 1 from Hanna Point [21].

Site selection prioritized ice-free areas with exposed sediment, absence of visible animal activity or contamination, and accessibility during the expedition period. At each location, the upper 5 cm of surface material was removed using sterile spatulas, and subsurface sediment (5–10 cm depth) was aseptically collected into sterile containers to minimize aerial contamination and access recently exposed substrate.

Collins Glacier samples were obtained from proglacial zones at the glacier–bedrock interface, including small subglacial cavities exposed by ongoing ice retreat. These sites represent recently deglaciated sediments with minimal post-exposure weathering, potentially harboring psychrophilic fungi that have adapted to cold and oligotrophic conditions. Deception Island samples were collected from volcanic soils in geothermally active areas, while Artigas and Hanna Point samples originated from coastal ice-free zones.

Samples were immediately placed in a cooler at 4 °C, transported to the Laboratory of Natural Products & Drug Discovery (NP&DD) at Universidad de La Frontera (Temuco, Chile), and stored at −20 °C until processing.

### 2.2. Sampling Site Characterization

In total, five samples were collected from Collins Glacier, two from Artigas base, eleven from Deception Island, and one from Hanna Point. The coordinates of the sampling place and the soil temperature are given in Table 1.

### 2.3. Isolation and Identification of Culturable Fungi from Antarctic Sediments

Fungi were isolated from sediments according to the protocol described by Gonçalves et al. (2016) [22]. In summary, 1 g of sediment was added to 9 mL of sterile saline solution (0.85% NaCl) and vortexed, then 1 mL of the supernatant was diluted by 10 and 100 times with saline solution. Then, 100 µL of each dissolution was spread onto a Petri dish with agar YM media (0.3% yeast extract, 0.3% malt extract, 0.5% peptone, 2% glucose, 2% agar, pH 6.2 ± 2) in triplicate. Plates were supplemented with chloramphenicol (100 μg mL^−1^). The plates were incubated at 15 °C for 30 days; the cultures were then isolated and grown again to obtain pure cultures.

Total genomic DNA was extracted from fresh cultures using the E.Z.N.A. Fungal DNA Mini Kit (Omega Bio-tek, Norcross, GA, USA) following the manufacturer’s protocols. The mycelium was ground into a fine powder by placing the tissue in a 2.0 mL screw-cap tube containing one 3.0 mm tungsten carbide bead, then shaking the tube in a Mini-BeadBeater-16 (BioSpec Products, Bartlesville, OK, USA) for 40 s at 3450 rpm.

For amplification of the internal transcribed spacer (ITS) region, the primers ITS1F [23] and ITS4 [24] were used. For the PCR reaction, GoTaq^®^ G2 Colorless Master Mix (Promega, Santiago, Chile) was used, according to the manufacturer’s instructions for a PCR reaction. In brief, we used 12.5 µL GoTaq^®^ G2 Colorless Master Mix (GoTaq^®^ G2 DNA Polymerase, 2x Colorless GoTaq^®^ G2 Reaction Buffer (pH 8.5), 400 μM dATP, 400 μM dGTP, 400 μM dCTP, 400 μM dTTP and 3 mM MgCl_2_), 9.5 µL nuclease-free water, 1 µL (10 µM) forward primer, 1 µL (10 µM) reverse primer, and 1 µL (25.5–862 ng/µL) DNA template. A touchdown PCR program was employed, with an initial denaturation step at 94 °C for 3 min, followed by 10 cycles of denaturation at 94 °C for 30 s, with annealing temperatures starting at 60 °C for 45 s (decreasing by 1 °C per cycle), and elongation at 72 °C for 1 min 15 s. This was followed by 26 cycles of denaturation at 94 °C for 30 s, annealing at 50 °C for 45 s, elongation at 72 °C for 1 min 15 s, and a final extension at 72 °C for 7 min. Sequencing was performed at the AUSTRAL-omics Core Facility (Faculty of Science, Universidad Austral de Chile; https://australomics.cl/, acceded on 5 June 2025). Forward and reverse sequence chromatograms were assembled and edited using Geneious Prime 2024.

The ITS consensus sequences were submitted to BLAST against sequences available in GenBank [25] (accessed online https://www.ncbi.nlm.nih.gov/genbank/ on 2 June 2025). To infer phylogenetic relationships, the sequences were analyzed with those from the general *Geomyces* and *Pseudogymnoascus* from the studies of Minnis & Lindner (2013) and Villanueva et al. (2021) [26,27]. This dataset was aligned using MAFFT v7.490 [28] with the E-INS-i option [29,30]. Maximum likelihood (ML) phylogeny was inferred using RAxML version 7.0.3 [31] under the General Time Reversible (GTR) model [32], with gamma-distributed rate variation across sites and a proportion of invariable sites (GTR+G+I). To evaluate support for nodes, bootstrap pseudo replicates were performed 1000 times using the fast-bootstrapping option. The graphical representation of the tree, with bootstrap values, was generated using FigTree 1.4.4 [33].

### 2.4. Fungal Culture and Total Extract Production

Fungal strains were cultured in 1000 mL conical flasks containing a semi solid media composed of 400 mL of YM broth and 10 g of cotton, for 21 days at 15 °C in static conditions. Extraction of the secondary metabolites from whole cultures was performed by removing the cotton (with mycelia) and the culture media. To the liquid phase, 400 mL of ethyl acetate (EtOAc) was added and extracted three times, while the cotton with mycelia was sonicated with 400 mL of a mixture methanol: EtOAc (1:1 *^v^*/*_v_*) three times. The organic layers were pooled together and dried in a rotary evaporator (45 °C and 150 mbar), obtaining the total organic extract according to methods used in the laboratory NP&DD, which was stored at −20 °C until its use.

### 2.5. Antimicrobial Assays

The qualitative antimicrobial assays were performed against four fungal strains *Cryptococcus neoformans*, *Trichosporon* sp., *Candida albicans*, *Pichia kudriavzevii*, and two bacteria, *Escherichia coli* ATCC 25922 and *Staphylococcus aureus* ATCC 25930 by disk diffusion agar tests. The crude extracts were dissolved in dimethyl sulfoxide (DMSO) to reach 3 mg/mL stock solutions, then 10 µL were added on a filter paper disk of 7 mm in diameter (300 µg/disk). Disks were placed over Mueller Hinton agar plates inoculated with 200 µL of each pathogen at a concentration of 2.5–5.0 × 10^5^ UFC/mL and then incubated at 37 °C for 24 h in a humid chamber. The inhibition halos were measured with a caliper and compared to the positive controls fluconazole (antifungal) and streptomycin (antibiotic), both at 50 µg/disk. Disks with DMSO were used as negative controls [34].

The quantitative in vitro antifungal assay was performed in triplicate by a broth microdilution method following Clinical Laboratory Standard Institute (CLSI) recommendations according to the document M27A. In summary, the samples were dissolved in dimethyl sulfoxide (DMSO) stock solution at 1200 μg/mL and then diluted with sterile water in a work solution at 120 μg/mL. Compounds were evaluated in 96-well microplates between 60 and 6.25 μg/mL by serial dilutions in culture media (RPMI, 100 µL). The inoculum (100 µL) was adjusted to yield a cell concentration of 2.5–5.0 × 10^5^ UFC/mL. A microorganism growth control and one non-inoculated well were included to ensure medium sterility. The plates were incubated at 37 °C for 48 h in a humid chamber. Then, the growth in the positive control column and its absence in the negative control were visually verified. The MIC values were determined as the lowest concentrations of each compound capable of inhibiting microorganism growth by visual inspection compared with growth control. MIC values were determined by triplicate, and each assay was performed three times using different starting yeast. Fluconazole was used as a positive control at concentrations of 50, 25, 12.5, 6.25, 3.13, 1.56, and 0.78 µg/mL. All experiments were performed in triplicate [35].

### 2.6. GCMS Analysis of Total Extracts

The analysis of fungal secondary metabolites was performed following the method outlined by Iqbal et al. 2025 [36], with modifications. The dried fungal extracts were dissolved in acetone to reconstitute the organic compounds. The samples were vortexed and centrifuged at 11,180× *g* for 10 min, and the supernatant was filtered through a 0.22 μm PTFE syringe filter prior to analysis.

GC-MS analysis was conducted using a GC Shimadzu 2030 (Shimadzu, Kyoto, Japan). Compound separation was achieved using a DB-5MS capillary column (30 m × 0.25 mm i.d., 0.25 μm film thickness) with helium as the carrier gas at a constant flow rate of 1 mL/min. The injector temperature was maintained at 280 °C with a splitless injection mode. The oven temperature program was initiated at 60 °C (held for 2 min), then ramped at 5 °C/min to 280 °C, and held for 5 min, yielding a total run time of 51 min. The mass spectrometer operated in electron ionization (EI) mode at 70 eV, scanning a mass range of 35–550 *m*/*z*. Both the ion source and transfer line temperatures were set at 230 °C and 280 °C, respectively. Compound identification was performed by comparing mass spectra with the NIST (National Institute of Standards and Technology) mass spectral library, with matches accepted at ≥70% similarity index. Compounds were identified based on their retention times, mass spectra fragmentation patterns, and relative peak abundances [36].

## 3. Results

### 3.1. Isolation of Antarctic Fungal Strains

A total of 14 fungi strains from 5 different genera were isolated from soil samples collected in the South Shetland Islands in January 2020. From Collins Glacier, four fungal genera were identified as *Cladosporium* sp., *Penicillium* sp., *Pseudeurotium* sp., and *Pseudogymnoascus* sp.; *Cladosporium* sp. and *Pseudeurotium* sp. were only found in the samples taken from Collins Glacier.

Fungi of the genus *Penicillium* was found in 3 of 11 samples taken from Deception Island, and *Pseudogymnoascus* sp. was also found in the soil sample taken from Artigas Base, in one of the soil samples taken from Deception Island, and in the only sample taken from Hanna Point. This could suggest a wider distribution of the latter fungi in the South Shetland Islands. Finally, the genus *Oidiodendron* sp. was found only in isolated samples from Deception Island.

### 3.2. Identification of Antarctic Fungal Strains Assayed

The identification and phylogenetic affiliation of the samples was based on the sequencing of the ribosomal internal transcribed spacer 2 (ITS2) region and obtained by sequence comparison with the information available in the GenBank database. The results of the molecular identification of the fungal strains are shown in Table 2. Strain ECA57-01 corresponds to the genus *Cladosporium*, ECA57-09, ECA57-44, and ECA57-57 to *Oidiodendron* (100% identical), and strains ECA57-09, ECA57-25, ECA57-27, and ECA57-55 to *Penicillium* (100% identical). The strains ECA57-12 and ECA57-32 were 100% identical and correspond to *Pseudogymnoascus*. The strains ECA57-03 and ECA57-5 were identified as *Pseudogymnoascus* sp. and were 100% identical. These strains differed in 2 insertions/deletions from ECA57-61 (Table 2).

**Figure 2:**
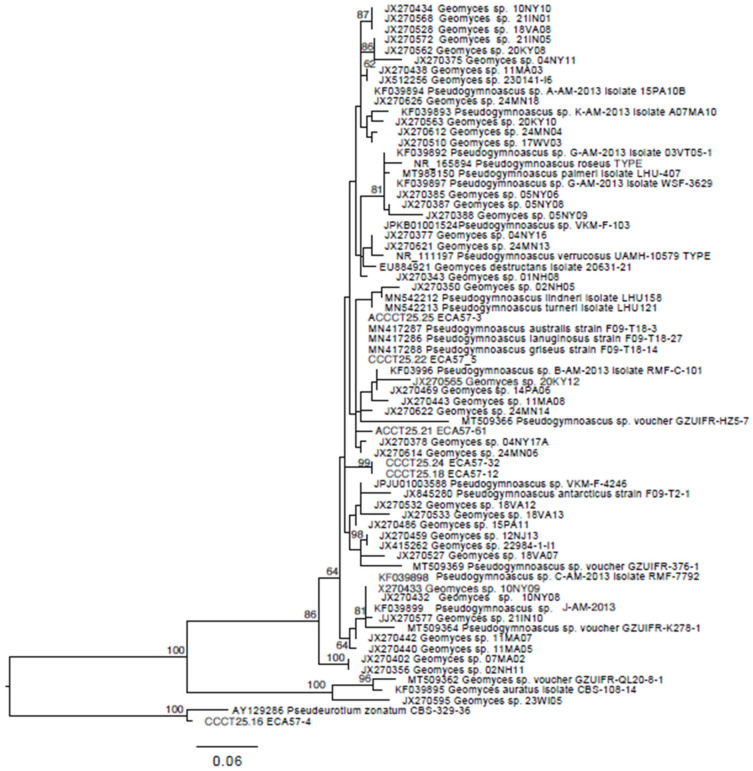
Phylogenetic relationships of fungal isolates based on internal transcribed spacer sequences belonging to the family Pseudeurotiaceae, inferred using RAxML maximum likelihood analysis. Bootstrap values are indicated above branches. *Pseudeurotium zonatum* CBS-329-36 and ECA57-4 were used as outgroups.

Based on our phylogenetic analysis, the strains ECA57-12 and ECA57-32 have an isolated position and seem to represent a new *Pseudogymnoascus* species (Figure 2). The strain ECA57-4 might represent a new species of *Pseudeurotium*. Sequences of the strains ECA57-03 and ECA57-5 are phylogenetically identical with MN417286 *Pseudogymnoascus lanuginosus*, MN417287 *G. australis*, and MN417288 *G. griseus*. The strains ECA57-61 had an isolated position, being closely related to the strains ECA57-03 and ECA57-5.

### 3.3. Antimicrobial Activity Assays

Five fungi extracts showed significant activity against at least one microorganism, displaying inhibitory halos >12 mm. Results are listed in Table 3.

The extracts of the Antarctic fungi ECA57-20 and ECA57-61 stood out due to their high antifungal activity with halos up to 24 and 27 mm against *Cryptococcus neoformans* and *Trichosporon* sp., respectively, Table 4. In addition, both showed activity against pathogenic yeasts of the *Candida* genus. ECA57-61 showed MIC above 7.81 µg/mL and CMF of 7.81 µg/mL against *C. lusitaniae.*

**Figure 3:**
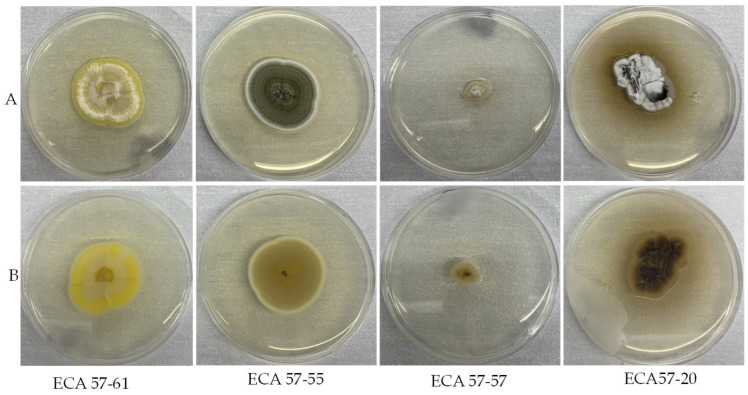
Images of fungi cultured in MH. agar for 21 days. Upper side (**A**) and reverse (**B**) of selected bioactive fungi *Pseudogymnoascus* sp. (ECA57-61), *Penicillium* sp. (ECA 57-55), *Oidiodendron* sp. (ECA 57-57), and *Oidiodendron* sp. (ECA57-20).

The culture of four active fungi are shown in Figure 3, where both strains of *Oidiodendron* presented different growth rate patterns.

### 3.4. Bioactive Composition Analysis Through GCMS

GC-MS metabolomic profiling was performed on total organic extracts from the five most bioactive Antarctic fungal strains to identify putative secondary metabolites that may contribute to the observed antimicrobial and cytotoxic activities (Table 5). A total of 28 compounds with NIST library similarity indices ≥70% were tentatively identified across these strains, with retention times ranging from 5.545 to 38.600 min.

The *Oidiodendron* sp. strain ECA57-20, which demonstrated strong anti-*Candida* activity (MIC 7.81–62.5 µg/mL), produced ethyl oleate and cridanimod as major metabolites. *Penicillium* sp. (ECA57-55), exhibiting antifungal properties, contained curcumin, ganaxolone, and several terpenoid derivatives. *Pseudogymnoascus* sp. (ECA57-61), the most potent antifungal strain (MIC 7.81 µg/mL against multiple *Candida* species), showed the highest chemical diversity with eleven putatively identified compounds including quinazoline derivatives (4(1H)-quinazolinone, 4-hydroxyquinazoline), cyclandelate, 5-hydroxyindole-3-acetic acid, fasoracetam, and aniracetam.

All compound identifications are tentative (MSI Level 3) and based on spectral library matching and require confirmation through authentic standards or multidimensional NMR analysis.

## 4. Discussion

*Oidiodendron* spp. have been identified in various Antarctic substrates, including macroalgae [37,38], freshwater [22], soils [39,40,41], sub-seafloor sediments [42], and permafrost [43,44]. Several strains of *Oidiodendron* spp. have been reported to produce bioactive metabolites [39,42,45]. In our study, fungi of codes ECA57-20, ECA57-44, and ECA57-57 from Deception Island were identified as *Oidiodendron* sp. The extract of ECA57-20 stood out in terms of its antifungal capacity, with halos between 18 and 24 mm in qualitative tests and MICs between 7.81 and 62.5 µg/mL against five different species of *Candida*. In addition, the extract showed a fungicidal capacity against three of these microorganisms, *P. kudriavzevii*, *C. tropicalis*, and *C. lucitaniae* with MICs of 250, 62.5, and 1000 µg/mL, respectively.

*Penicillium* spp. are among the most widespread fungi, inhabiting diverse environments worldwide and exerting significant economic and health impacts [46]. In Antarctica, they are commonly found in soil, snow, ice, seawater, marine and freshwater sediments, lakes, plants, and animals [44,47,48]. These fungi produce a wide range of secondary metabolites that vary according to species and environmental conditions [49]. Some *Penicillium* species are notable producers of the mycotoxins patulin and ochratoxin A with antibiotic properties [46,50]. Kozlovski et al. (2012) provides a comprehensive review of *Penicillium* metabolites with antimicrobial and antitumor activity [50]. In this study, the Antarctic fungi ECA57-09 (from Collins Glacier) and ECA57-25, ECA57-27, and ECA57-55 (from Deception Island) were identified as *Penicillium* species and showed minimal antimicrobial activity.

*Pseudeurotium* spp. have been isolated in Antarctica from lakes [51,52,53], glaciers [15], marine sediments [54,55], and soils [56,57]. From *Pseudeurotium zonatum* several bioactive metabolites have been reported, including cytochalasin G and three novel cytochalasins (X, Y, Z) [58]. More recently, Duan et al. (2024) identified ten new ketoglobosins, chaepseubakerins A–J, from *Pseudeurotium bakeri*, with chaepseubakerin A displaying potent cytotoxic activity against multiple human cancer cell lines and inducing G2/M arrest and apoptosis [59]. In this study, the strain ECA57-04, isolated from Collins Glacier that was identified as *Pseudeurotium* sp. showed minimal antimicrobial activity.

*Pseudogymnoascus* spp. are widely distributed in Antarctica, inhabiting diverse substrates such as soils [60], mosses, *Colobanthus quitensis* leaves [47], macroalgal thalli [37], freshwater lakes [22], marine sediments [48], and lichens. Despite their abundance, the knowledge about their metabolites remains limited [27]. Figueroa et al. (2015) identified four novel nitroaspartic acid derivatives (pseudogymnoascins A–C and 3-nitroaspartic acid) together chestin and pyriculamide [61]. Shi et al. (2021) isolated from *Pseudogymnoascus* sp., pseudophenone A and six known polyketides with antibacterial activity [62]. After that, they also identified a novel pyridine derivative, and eight diketopiperazines [62]. Antipova et al. (2023) reported the isolation of (+)-macrosphelides A and B from *Pseudogymnoascus* strains collected in Arctic soils, which exhibited antimicrobial and antitumor activity [63]. In this study, the Antarctic fungus ECA57-61, isolated from Hanna Point sediments and identified as *Pseudogymnoascus* sp., exhibited strong antifungal activity, with inhibition halos of 27 mm against *Trichosporon* sp. and 18–20 mm against *Candida albicans* and *P. kudriavzevii*. Quantitative assays showed a MIC of 77.8 µg/mL against *C. lucitaniae*.

The genus *Cladosporium* has been reported in various Antarctic environments, including lakes [64], rocks [65], snow, and air [66]. *Cladosporium* spp. are known producers of diverse secondary metabolites with biological activities [67,68]. However, the total extract from the Antarctic strain ECA57-01, identified as *Cladosporium* sp. and isolated from Collins Glacier soil, showed no activity against pathogenic microorganisms instead, its potential as a biotransforming agent was explored in the hydroxylation of drimane sesquiterpenoids with antifungal activity against candida yeast [35].

To contextualize the novelty of our bioactive isolates, we compared strains ECA57-20 and ECA57-61 with their closest phylogenetic relatives for which biological and chemical data are available. Strain ECA57-20 showed 95.55% ITS identity to *Oidiodendron truncatum*, falling below the commonly accepted 97–98% threshold for conspecific delimitation in fungi [69,70]. Previous studies on *O. truncatum* have reported the production of cytotoxic epipolythiodioxopiperazines (chetracins) and diketopiperazines [39], as well as antifungal tetranorditerpene lactones (oidiodendrolides) and norditerpene oidiolactones active against *Candida albicans* and *Cryptococcus neoformans* [71,72]. In contrast, our *Antarctic Oidiodendron* sp. ECA57-20 exhibited a distinct GC-MS metabolite profile and, more importantly, a broad anti-Candida spectrum (MIC 7.81–62.5 µg/mL) against five pathogenic species, including *C. albicans*, *Pichia kudriavzevii*, *C. tropicalis*, *Nakaseomyces glabratus*, and *Clavispora lusitaniae*. This activity profile against multiple non-albicans *Candida* species has not been previously demonstrated for this genus, suggesting that ECA57-20 may possess unique biosynthetic capabilities shaped by Antarctic environmental adaptations.

Similarly, ECA57-61 showed 98.59% ITS identity to *Pseudogymnoascus hyalinus*, near the species boundary threshold. While *Pseudogymnoascus* spp. are recognized as prolific decomposers in Antarctic ecosystems and producers of diverse metabolites including asterric acid derivatives [61] and polyketides [73], specific anti-Candida activity has not been reported for *P. hyalinus* or related species. Recent studies on *P. hyalinus* from deep-sea environments have focused on ferroptosis inhibition [74] rather than antifungal activity. Our isolate ECA57-61 demonstrated potent anti-Candida activity (MIC 7.81 µg/mL against *C. albicans*, *P. kudriavzevii*, and *N. glabratus*) alongside a chemically diverse GC-MS profile including quinazoline-type compounds, which are not typically associated with this genus. This potent antifungal activity against clinically relevant Candida pathogens, combined with the distinct metabolite profile, positions ECA57-61 as a particularly novel bioprospecting find. The distinct metabolite profiles and bioactivities of both ECA57-20 and ECA57-61, compared to their closest genetic relatives, indicate that the extreme Antarctic environment of the South Shetland Islands may act as a driver for the evolution of unique secondary metabolic pathways with therapeutic potential.

GC-MS metabolomic profiling of the five most bioactive Antarctic fungal strains revealed chemically diverse secondary metabolite profiles that may partially explain the observed antimicrobial and cytotoxic activities, though all identifications remain putative (MSI Level 3) pending confirmation. Notably, cryptolepine detected in *Oidiodendron* sp. (ECA57-57) is an indoloquinoline alkaloid with documented antimicrobial and cytotoxic properties [75,76]. The presence of quinazoline derivatives in *Pseudogymnoascus* sp. (ECA57-61) aligns with its potent antifungal activity (MIC 7.81 µg/mL), as this scaffold appears in known antimicrobial agents. However, critical caveats apply many “pharmaceutical” compounds identified through NIST library matching (ganaxolone, fasoracetam, aniracetam) likely represent structurally similar fungal natural products rather than actual synthetic drugs, as these matches reflect shared functional groups or carbon skeletons, not molecular identity. Furthermore, the observed bioactivities result from complex mixtures; attributing effects to individual components without isolation and bioassay-guided testing remains speculative. The distinct metabolite profiles observed even among congeneric strains (*Oidiodendron* ECA57-20, -44, -57) despite taxonomic similarity underscore the influence of genetic variability and microenvironmental adaptation on secondary metabolism in Antarctic fungi. This preliminary chemical profiling effectively prioritizes strains ECA57-20, ECA57-55, ECA57-57, and ECA57-61 for scale-up fermentation, bioassay-guided fractionation, and definitive structural elucidation via multidimensional NMR and HRMS to identify the specific molecules responsible for the antimicrobial and cytotoxic activities.

The metabolic diversity observed among strains of the same genus underscores the influence of microenvironmental factors and genetic variability on secondary metabolite production in Antarctic fungi. The *Pseudogymnoascus* strains, despite taxonomic similarity, exhibited distinct chemical profiles: ECA57-61 from Hanna Point showed the highest metabolite diversity and strongest antifungal activity, while ECA57-3 and ECA57-5 from Collins Glacier and Deception Island displayed different compound profiles with cytotoxic rather than antimicrobial properties. This metabolic plasticity may reflect adaptive responses to varying Antarctic microhabitats and suggests that comprehensive chemical profiling of multiple strains, even within the same genus, is essential for bioprospecting efforts. The identification of multiple nootropic compounds (fasoracetam, aniracetam) and pharmaceutical agents (cyclandelate, salsalate) in these extracts warrants further investigation through bioassay-guided fractionation to isolate and characterize the specific bioactive principles responsible for the observed pharmacological activities.

## 5. Conclusions

This study demonstrates that Antarctic fungi from the South Shetland Islands produce bioactive metabolites with significant potential as alternative antifungal agents. The *Oidiodendron* sp. (ECA57-20) and *Pseudogymnoascus* sp. (ECA57-61) strains exhibited strong activity against multiple *Candida* species, with MIC values ranging from 7.81 to 62.5 µg/mL, addressing the urgent need for new therapeutic options against drug-resistant fungal pathogens. GC-MS analysis revealed diverse secondary metabolites including farnesol, cryptolepine, ganaxolone, and other pharmacologically relevant compounds that may account for the observed biological activities. These findings highlight the value of Antarctic ecosystems as unexplored sources of novel bioactive compounds and warrant further investigation through bioassay-guided fractionation to isolate and characterize the specific molecules responsible for the antimicrobial and cytotoxic effects. Future studies should focus on elucidating mechanisms of action, evaluating toxicity profiles, and assessing therapeutic potential to advance these discoveries toward practical applications in treating fungal infections.

## Figures and Tables

**Table 1:** South Shetland sampling sites description.

Sample	Localization	Coordinates	T °C
1	Collins Glacier A	S 62°10′07″ W 58°51′00″	1.4
2	Collins Glacier B	S 62°09′46″ W 58°50′53″	1.2
3	Collins Glacier C	S 62°09′35″ W 58°49′32″	0.6
4	Collins Glacier E	S 62°10′18″ W 58°54′57″	0.6
5	Collins Glacier F	S 62°10′07″ W 58°55′08″	1.1
6	Artigas A	S 62°11′09″ W 58°54′13″	1.4
7	Artigas B	S 62°10′59″ W 58°53′31″	1.4
8	Deception Island A	S 62°56′11″ W 60°44′13″	1.2
9	Deception Island B	S 62°59′43″ W 60°36′23″	1.2
10	Deception Island C	S 62°59′34″ W 60°36′49″	1.3
11	Deception Island E	S 62°59′40″ W 60°37′45″	1.4
12	Deception Island F	S 62°59′28″ W 60°38′14″	1.4
13	Deception Island H	S 62°57′41″ W 60°43′09″	1.8
14	Deception Island I	S 62°55′57″ W 60°35′50″	1.8
15	Deception Island J	S 62°58′15″ W 60°35′11″	1.5
16	Deception Island K	S 62°58′21″ W 60°34′40″	1.4
17	Deception Island L	S 62°58′28″ W 60°34′14″	1.3
18	Deception Island M	S 62°58′42″ W 60°33′58″	3.4
19	Hanna Point A	S 62°33′48″ W 60°41′26″	1.2

**Table 2:** Molecular identification of fungal isolates from Antarctic samples using BLAST searches.

Strain	CCCT Deposit Number	Top BLAST Search Results (GenBank Accession Number)	Query Cover (%)	Identity (%)	Number of bp Analyzed	Proposed Taxa (GenBank Acc. No.)
ECA57-01	CCCT25.14	*Cladosporium macrocarpum* CBS:121623 NR_119657	100%	100%	564	*Cladosporium* sp. PV719319
ECA57-20	CCCT25.23	*Oidiodendron truncatum* UAMH 1399 NR_111036	91%	95.55%	525	*Oidiodendron* sp. PV719325
ECA57-44	CCCT25.19	*Oidiodendron truncatum* UAMH 1399 NR_111036	91%	95.55%	525	*Oidiodendron* sp. PV719329
ECA57-57	CCCT25.20	*Oidiodendron truncatum* UAMH 1399 NR_111036	91%	95.55%	525	*Oidiodendron* sp. PV719331
ECA57-09	CCCT25.17	*Penicillium crustosum* FRR 1669 NR_077153	100%	100%	610	*Penicillium* sp. PV719323
ECA57-25	CCCT25.07	*Penicillium crustosum* FRR 1669 NR_077153	100%	100%	610	*Penicillium* sp. PV719326
ECA57-27	CCCT25.08	*Penicillium crustosum* FRR 1669 NR_077153	100%	100%	610	*Penicillium* sp. PV719327
ECA57-55	CCCT25.09	*Penicillium crustosum* FRR 1669 NR_077153	100%	100%	610	*Penicillium* sp. PV719330
ECA57-04 *	CCCT25.16	*Pseudeurotium bakeri* CBS 878.71 NR_145345	90%	99.41%	505	*Pseudeurotium* sp. PV719321
ECA57-12 *	CCCT25.18	*Pseudogymnoascus hyalinus* CBS 106.13 NR_190914	98%	98.04%	641	*Pseudogymnoascus* sp. PV719324
ECA57-32 *	CCCT25.24	*Pseudogymnoascus hyalinus* CBS 106.13 NR_190914	98%	98.04%	641	*Pseudogymnoascus* sp. PV719328
ECA57-03 *	CCCT25.15	*Pseudogymnoascus hyalinus* CBS 106.13 NR_190914	98%	97.88%	641	*Pseudogymnoascus* sp. PV719320
ECA57-05 *	CCCT25.22	*Pseudogymnoascus hyalinus* CBS 106.13 NR_190914	98%	97.88%	641	*Pseudogymnoascus* sp. PV719322
ECA57-61 *	CCCT25.21	*Pseudogymnoascus hyalinus* CBS 106.13 NR_190914	98%	98.59	641	*Pseudogymnoascus* sp. PV719332

* Fungal strains submitted to phylogenetic analysis. Samples numbers correspond to the positions were collected according to Table 1.

**Table 3:** Antimicrobial activity formatting of mathematical components.

Isolate	Diameter of Growth Inhibition (mm)					
	Antifungal Activity				Antibacterial Activity	
	*Cryptococcus neoformans*	*Trichosporon* sp.	*Candida* *albicans*	*Pichia kudriavzevii*	*Staphylococcus* *aureus*	*Escherichia* *coli*
ECA 57-3	-	-	10	-	-	-
ECA 57-4	10	10	10	-	-	-
ECA 57-5	10	11	11	-	-	-
ECA 57-9	-	13	9	-	-	-
ECA 57-20	24	18	18	20	11	8
ECA 57-27	9	-	8	-	-	-
ECA 57-32	9	-	9	9	-	-
ECA 57-44	14	-	-	-	17	12
ECA 57-55	-	-	-	-	14	-
ECA 57-57	11	-	-	-	18	11
ECA 57-61	15	27	18	20	16	15

The average results derived from triplicate assays are shown as the diameter of growth inhibition (mm) for all the pathogens tested. The negative results are shown with (-). Only active fungi extracts are shown.

**Table 4:** Quantitative activity against *Candida* yeast for ECA57-20 and ECA57-61. Results in µg/mL.

Yeast	ECA57-20		ECA57-61		Fluconazole	
	MIC	MFC	MIC	MFC	MIC	MFC
*C. albicans*	62.5	-	7.81	15.6	0.8	0.8
*Pichia kudriavzevii*	31.25	250	7.81	15.6	12.5	12.5
*C. tropicalis*	31.25	62.5	31.25	62.5	0.8	0.8
*C. glabrata*	7.81	-	7.81	62.5	3.13	3.13
*C. lusitaniae*	31.25	1000	-	7.81	0.8	0.8

**Table 5:** Bioactive compounds identified in fungal extracts by GC-MS analysis.

Strains	RT (min)	SI (%)	Compound Name	PubChem ID	Molecular Formula	Molecular Weight (g/mol)
ECA57-20	23.240	94	Ethyl Oleate	5363269	C_20_H_38_O_2_	310
	35.955	70	Cridanimod	38072	C_15_H_11_NO_3_	253
ECA57-44	5.545	86	alpha-Hydroxyisocaproic acid	92779	C_6_H_12_O_3_	132
	6.575	80	Cumene hydroperoxide	6629	C_9_H_12_O_2_	152
	7.070	96	4-methyl-2-oxopentanoic acid	70	C_6_H_10_O_3_	130
	9.460	76	5-Chloro-1-pentanol	78915	C_5_H_11_ClO	122
ECA57-55	7.495	75	Isopulegol acetate	1549026	C_12_H_20_O_2_	196
	8.845	90	Butanal	16331	C_9_H_16_O_3_	172
	16.655	87	Sedoheptulose	5459879	C_7_H_14_O_7_	210
	17.575	70	Curcumin	969516	C_21_H_20_O_6_	368
	34.970	79	2-Dodecen-1-yl (−) succinic anhydride	5367549	C_16_H_26_O_3_	266
	37.350	91	Ganaxolone	6918305	C_22_H_36_O_2_	332
ECA57-57	20.415	75	Geranyl tiglate	5367785	C_15_H_24_O_2_	236
	29.620	80	*N*-(4-Hydroxyphenylacetyl) piperidin-2-one	1370	C_13_H_15_NO_3_	233
	34.740	79	Beta-caryophyllene	5281515	C_15_H_24_	204
	36.795	85	Cryptolepine	82143	C_16_H_12_N_2_	232
	38.600	70	Asteltoxin	6438150	C_23_H_30_O_7_	418
ECA57-61	14.945	73	4(1H)-Quinazolinone		C_8_H_6_N_2_O	146
	15.795	93	4-Hydroxyquinazoline	135408753	C_7_H_10_N_2_O_2_	154
	16.075	77	2-(2,4-dichlorophenoxy)-5-ethylphenol	25023958	C_14_H_12_Cl_2_O_2_	282
	16.740	88	Fasoracetam	198695	C_10_H_16_N_2_O_2_	196
	18.325	79	Cyclandelate	2893	C_17_H_24_O_3_	276
	20.245	83	5-Hydroxyindole-3-acetic acid	1826	C_10_H_9_NO_3_	191
	21.000	70	Impentamine	9793868	C_8_H_15_N_3_	153
	22.210	80	Aniracetam	2196	C_12_H_13_NO_3_	219
	28.285	72	1-(4-methoxy-6-methylquinazolin-2-yl) guanidine	600062	C_11_H_13_N_5_O	231
	29.255	70	Salsalate	5161	C_14_H_10_O_5_	258
	29.945	71	Iadademstat	71543365	C_15_H_22_N_2_	230

## Data Availability

The original contributions presented in this study are included in the article. Further inquiries can be directed to the corresponding authors.
